# Peripheral Nerve Lesions

**Published:** 1918-08

**Authors:** W. L. Crosthwait

**Affiliations:** Waco, Texas


### Peripheral Nerve Lesions.

W. L. CROSTHWAIT, M. D., WACO, TEXAS

We will briefly consider this subject under three general subdivisions: (a) Symptomatology, (b) Prognosis and (c) Treatment. However, we will preface our discussion by stating a few things of fundamental importance relating to the degeneration and regeneration of peripheral nerves which are now definitely established.

The war has undoubtedly given a greater impetus to the study and research work in neurological surgery and the peripheral nerves have come in for the most thoughtful consideration so that several questions which heretofore were much debated have become settled.

First is nerve degeneration. When a nerve is cut the peripheral end degenerates to the proximal cell. This process of degeneration begins soon after the nerve is divided. The first step is the breaking up of the medulary sheath into small masses or droplets of myelin which slowly absorb. This process is followed by a similar degeneration of the axis-cylinder which likewise becomes broken up and absorbed. This process takes place simultaneously throughout the entire length of the nerve distal to the section and all that is left of the nerve is the neurilemma or neucleated sheath of Schann, the neuclii of which are greatly increased. Thus we see nature's wise provision to take care of the regeneration of a divided nerve under proper conditions in the due course of time. In passing we might add that the same process of degeneration occurs when a nerve is divided regardless of the time of suturing whether immediate or remote. Also it must be remembered that the axis-cylinder degenerates to its proximal cell regardless of the site of lesion. In other words if a sensory axis-cylinder is cut the proximal end will degenerate to the cell in the intervertebral ganglion on the posterior spinal root, and when a motor axis-cylinder is cut degeneration will likewise take place to its cell in the anterior horn. Just what further phenomena of ascending degeneration takes place is not definitely settled.

(Microsopically we observe in a peripheral nerve which is divided that a bulbous formation will form on the proximal end, some times called a neuroma and that a much smaller formation will be noted on the distal end, or both ends, or the proximal and distal neuromas will usually be connected or united in scar tissue connecting them to the surrounding structures, the entire mass being sometimes described as a neuroma, (which name is incorrect.)

The physiological phenomena of divided nerves are briefly as follows: (1) Motor; (a) Atrophy of the paralyzed muscle, (b) reduced irritability; by the seventh day they cease to respond to the Fardic current, by the tenth day a characteristic change in their response to the Galvanic current; (c) complete atrophy of muscle and the characteristic fibros. (2) Sensory, (a) anesthesia of parts supplied, cutaneous, muscle, tendon, bone and joint, due consideration being given to nerve overlapping and to Sherington's law of each sensory area being influenced by three nerve roots; (b) analgesia of parts supplied of course with the same observation as above.

(3) Trophic, (a) skin, note the tardy desquimation of the insensitive areas, the scales adhering to the surface, etc., cutaneous ulceration, atrophy of sweat glands, (b) due to trophic disturbance the muscles atrophy unless nutrition is maintained by massage and electrical treatment. (This is of the greatest importance, as the failure to recognize this fact in the past has been the cause of much disappointment in nerve surgery.)

The second question before passing to the main consideration of our subject is nerve regeneration. To this we refer very briefly.

(a) After suturing the ends of a divided nerve the various elements in sensibility return in a definite order; sensibility to deep pressure first, followed by the recognition of painful cutabeous stimuli (protopathic) and lastly by the sensibility to light cutaneous impressions (epicritic.) About the time of the appearance of the finer sensibility to the three fundamental or rather elemental responses to touch, temperature and pain, motor power begins to return, voluntary motion being observed in the muscles nearest the seat of lesion. By some authors it has been claimed that motor fibres are more apt to regenerate and some have claimed that voluntary motion should precede sensation. This has not been the experience of the recent research workers and observers.

(b) As to structure, at the end of three weeks new axis-cylinders are formed within the neurolemma sheaths by the deposition of a thin thread along the side of the spindle called neurolemma cell, these fibres grow from each end of this cell until they meet those growing out from the cells above and below. Perhaps the best reference on this most interesting phase of this subject is the experimental work of Drs. Edwin G. Kirk and Dean Lewis on regeneration of peripheral nerves in the Morris Institute of Medical Research of the Michael Reese Hospital. John Hopkins Hospital Bulletin, Vol. 28, No. 312. Feb., 1917.

The new medullary sheath makes its appearance about the fourth week. This, too, has been shown to be a projectile process from the neurolemma cell. It is interesting to observe from day to day in the experimental study of divided and resutured nerves how these projections from both the proximal and distal ends will seemingly feel around for each other. In passing we might say that it is this effort of nature to unite a divided nerve that causes the formation of the so-called neuromas. It must be admitted that the same process of regeneration takes place whether the ends of the divided nerve are sutured or not. However, it must be perfectly clear that if the ends are allowed to remain separated that these regenerating axis-cylinders and medullary sheaths must be more tardy in their appearance and more apt to become lost and entangled in intervening scar tissue, while the reverse must be true when either primary or scondary suture is properly performed.

We pass now to the main consideration of our subject.

The symptomatology of peripheral nerve lesions must be studied in connection with character of injury and the manner of their production.

The symptoms of nerve injury may occur: (1) Immediately upon receipt of trauma, (2) at a variously remote time but consequent upon trauma and (3) independent of any known trauma.

We may expect immediate symptoms to follow a peripheral nerve lesion when the nerve is either completely divided, torn, contused, compressed and concussed.

Symptoms occurring subsequent to trauma but consequent upon it are due to either the involvent of the nerve in scar tissue, or callus.

Symptoms occurring independent of any known trauma are due to compression from new growths, cervical ribs and toxic conditions.

We need not dwell upon the fact that if a nerve is completely divided that there will be complete loss of sensation and loss of motion of the muscles supplied by it below the point of lesion.

It is the case of the partial or incomplete division of the nerve that we find a complex symptomatology. In practically all cases there is loss of epicritic sensibility. There is usually partial paralysis of all the muscles or complete paralysis of some of the muscles supplied with little difference to reaction of the others. Upon this one point may rest the differential diagnosis between complete and partial division of the nerve. In some cases there is loss of the finer sensibilities with no motor disturbance, while in other cases pain and tenderness over the course of the nerve is the predominant feature. It is said that in the nerve lesions, incomplete divisions, due to high power rifle bullets, that the pain is very intense and immediate and is of a burning character. The term employed during the Civil War by Dr. Wier Mitchell, *Causalgia,* a burning pain, is again coming into use to describe the condition of pain which follows many cases of partial nerve lesions, and which might just as appropriately be described as painful interstitial neuritis, and in such cases where operations have been performed, the nerve has been found to be incompletely divided and wrappd in scar tissue.

For several days after a nerve injury it may be impossible to say whether the nerve is completely divided or not. There may be complete loss of both motion and sensation in incomplete divisions of the nerve, but after say ten days, in the cases of incomplete division there will be the so-called reaction of incomplete division (Sherrin, Herrick, Bassoe and Tinel), that is there will be no reaction to the faradic current but reaction to the galvanic current, but much weaker than on the normal side. In practice all one needs to make this test is the small galvanic battery with close observation of the reaction of the normal opposite muscle, compared with the injured one.

In torn or lacerated nerves the lesion is usually produced by traction on the limb. The brachila plexus or some of its component nerve roots is the most common site of injury. A nerve may be streached to the point of breaking, the sheath is usually the first to give way, even a complete breaking of the nerve sheath may result in only a partial division of the nerve fibers or funiculi and therefore the symptoms which follow may be either those of complete or incomplete nerve division as pointed out above.

A contused nerve is one that suffers loss of function due to trauma applied directly to the nerve or over it when such trauma does not result to anatomical division. Nerve contusion is what has happened to many nerves in the attempted repair by improper technic. The symptoms of nerve contusion will vary according to the extent of the injury. A contusion may amount almost to a complete crushing of the nerve with symptoms of complete division.

In other cases only a few nerve fibres may be involved giving rise to a very meagre symptomatology.

By nerve compression is meant injuries produced by pressure slowly and steadily applied. Such injuries often result from improperly applied splints, illy applied bandages, crutch pressure (the musclo-spiral often injured). Also sleep and anesthesia compression. Numbness and tingling with a heavy feeling in the limb is the usual syndrome in the mild cases. If the pressure is continuously applied it may result in such degenerative changes as will amount to a complete division of the nerve.

Nerve concussion it may be said is a war term. A nerve is said to be concussed when its conductivity is impaired by trauma near it. Such condition of nerves is observed where high power rifle bullets pass near a nerve trunk. There is no direct injury to the nerve from a miscroscopical standpoint. There is undoubtedly a miscroscopical change in the nerve trunk comparable to that which takes place in the brain in brain contussion. Symptoms of paralysis follow immediately, but upon careful examination it will be found that the paralysis is not complete, that the loss of sensation is partial and transient and that the muscles have retained their normal electrical reaction. This is the main diagnostic point.

In cases following injuries of such a nature and location as might well result in a nerve lesion, but where there is no evidence of a nerve injury at the time or immediately following the injury, and where such symptoms show up some weeks or months later, we may conclude that we have involvement of the nerve by scar tissue or bone callus. Such symptoms may come on slowly and gradually progress to those of complete division of the nerve. It has been shown in this war that this is an exceedingly common occurrence. By way of digression we might add that the best management of such injuries is in their prevention, which can be done by the correct management of the primary injury, the judicious massage of limbs, passive motion, the prevention of infection and the hastening of blood clots, etc.

### Prognosis.

The very long time required for nerve regeneration has caused much discouragement in nerve surgery on the part of both surgeon and patients.

The time of complete regeneration, of course, will vary under the many conditions met with; it is quicker in primary suture than secondary; it varies with different nerves, and finally with the after management.

Complete regeneration may never take place but appreciable regeneration will occur in sensory nerves in from two to three years and in motor nerves in one to two years. In mixed nerves, motion will being to appear in the second year and sensation will be restored during the third year. An operator should not despair of success until this time has passed.

In primary suture under septic condition and with correct after treatment we may expect the following phenomena:

Protopathic sensation will commence within six weeks; will be fairly well established within six months.

Epicritic sensation will commence within six months and will be well nigh complete within twelve months.

Motor power will being within six months and will be fully established in twelve months.

In secondary suture under the same conditions the sensations will return in the same order but may require twice the time or even longer.

In all septic wounds the time and manner of return of sensation and motion will vary within wide limits.

### Treatment.

The treatment of peripheral nerve lesions is operative or active and non-operative or expectant.

Operative treatment may be classified as primary or immediate and secondary or remote.

The factors which determine success are early and accurate diagnosis, correct anatomical approximation, the maintenance of nutrition and relaxation of parts supplied by the injured nerve.

Of course it is perfectly clear that the treatment of a divided peripheral nerve is suture and as primary suture gives the quickest and surest results it is always desirable and unquestionably the operation of choice. However, there are many valid reasons why primary suture is not performed; (1) diagnosis is not quite absolute even in such lesions as might well be calculated to produce a decision of a nerve trunk, there is always a doubt unless the divided ends of the nerves are actually seen. In projectile wounds a divided nerve is not usually seen, unless the wound is enlarged for the purpose of controlling hemorrhage, etc. Of course in incised or contused wounds over the course of nerve trunks due attention should be given to the adequate repair of injured nerves in the primary suture of the wounds. If a wound is opened up in the primary treatment for the suture of a tendon or management of a bone fracture attention should be directed to the nerve.

Primary suture must be done by a wide or open exposure. It is a grave techincal error to go blindly feeling for a nerve in a lesion of any kind. Nerves are often further injured in blindly catching bleeding vessels.

Primary suture is desirable if there is a reasonable chance that the wound is sterile. The primary essential for the success of nerve suture is asepsis. Most punctured or projectile wounds are septic from the first and practically all war wounds are septic after eight hours.

Thus it will be seen that comparatively few nerve divisions are given primary treatment.

The adquate management of infected wounds with nerve lesions is the treatment of the infection; no attention should be paid to the divided nerve in the presence of infection.

When infection has subsided and the consequent scar tissue has reached a condition favorable secondary suture may be performed with just as good ultimate results.

As soon as a nerve is divided the muscles supplied by it begins to undergo trophic changes, they begin to streach out and become relaxed and elongated, the tendons undergo like changes and also become adherent in their sheaths. Therefore when a nerve division is apparent or suspected, during the period awaiting secondary repair attention must be directed to the muscles and tendons, to the end that the paralyzed muscles are not allowed to stretch and they must be made to frequently contract, and the tendons must be made to move in their sheaths. Manual and electrical massage is essential. The failure to take care of the paralyzed parts is sure to invite disappointment in the contemplated secondary suture. The joints must be given passive motion and a paralyzed joint must not be allowed to remain in a fixed position.

We will not enter at length into the non-operative or expectant treatment of injured nerve lesions; however, it must be known that many nerve lesions, even complete divisions ultimately recover without suture.

A limb whose nerve has been injured should be carefully protected against cold, pressure, fixational positions unfavorable to recovery, etc. The tendency of the antagonist muscles to pull in the opposite direction must be considered. The drop wrist should be dorsi flexed and the drop foot in injuries to the sciatics must be kept at right angles. This same principle must be carried relative to all other parts affected by nerve injury.

Several new points have been emphasized in the operative technic. We will briefly refer to the principal ones. The first is absolute asepsis. If possible more caution must be taken in nerve surgery than we have been wont to take in our bone work. The manner of exposing the nerve is next important. A good knowledge of the anatomy of the parts is essential. The approach to the nerve must be an anatomical approach. The nerve trunk should not be disturbed in situ but the surrounding tissue should be desected away from its sheath. The neuromas should be carefully lifted up and cut squarely off with a very sharp knife. The nerve trunk positively must not be touched only in the manner described. With small mouth tooth forceps the nerve sheath is caught up and held in place for suturing. It would be a fatal error in technic to catch the nerve trunk with a forceps like one would grasp an artery or bleeding vessel. All hemastacis must be controlled. A dry field is essential for careful and correct technic in operation and for favorable union of the sutured nerve.

The manner of union of divided nerves is end to end suture through the nerve sheaths. The suture material used is either fine plain cat-gut or silk. The sutures are placed by holding the divided nerves by catching the sheath of same with small mouth tooth forcep sin such a way as not to injure any of the nerve faciculi and in such a manner as to bring the nerve ends into apposition as they were before severance.

The prevention of adhesions and the formation of scar tissue is quite a problem and much experimental work has been done along that line. Perhaps fascia taken from the thigh with the smooth surface turned in is the best material to enclose the nerve at the point of union. Fat is sometimes used.

The hardest problem is where there is a considerable gap between the divided nerve to bridge. Just how far this bridging process may be successful is now the object of much experimental work. Different methods are now being used. The English are using gelatin tubes, the Americans are using fascia as described above, the French have used fat and pieces of veins. The object is to provide a trough along which the nerve axones with their neurolema sheaths may grow uninterrupted by scar tissue or other intervening tissue.

It has been shown that under favorable conditions such a nerve as the musulospiral will bridge a gap of two inches.

We will in a later paper speak further of this point and also discuss nerve mixing or switching and nerve implantation.

